# Neonatal screening for congenital cytomegalovirus infection in Tehran, Iran, using Guthrie cards

**Published:** 2020-06

**Authors:** Samileh Noorbakhsh, Mohammad Farhadi, Faezeh Haghighi, Sara Minaeian, Morteza Haghighi Hasanabad

**Affiliations:** 1 Pediatric Infectious Diseases Research Center, Institute of Immunology and Infectious Diseases, Iran University of Medical Sciences, Tehran, Iran; 2 Head and Neck Research Center, Iran University of Medical Sciences, Tehran, Iran; 3 Cellular and Molecular Research Center, Sabzevar University of Medical Sciences, Sabzevar, Iran

**Keywords:** Screening, Congenital *cytomegalovirus* infection, Guthrie card

## Abstract

**Background and Objectives:**

Cytomegalovirus (CMV) constitutes the most common viral cause of congenital infections in newborns worldwide. There are a significant number of asymptomatic newborns with congenital CMV infection in Iran, which may develop long-term sequelae of infection. Unfortunately, limited data exsists from Iran on the rate of congenital CMV infection among neonates. The current study was aimed to investigate the prevalence of congenital CMV infection among Iranian neonates by testing Guthrie cards.

**Materials and Methods:**

Guthrie cards were collected from infants within 2 weeks of life, and total DNA was extracted from samples by thermal shock and evaluated for CMV DNA using nested-PCR assay. CMV infection in newborns was confirmed through a commercial CMV PCR kit. Infected infants underwent further evaluation at the hospital.

**Results:**

CMV infection was identified in four of 1174 infants (0.34%) which is approximately 3 cases per 1000 live births. Infected infants were asymptomatic at birth and had a normal hearing status similar to other children. There were no factors in relation with CMV infection among newborns.

**Conclusion:**

According to the results of this study, infected infants with congenital CMV infection could identify at early stage by testing Guthrie cards (within 21 days of life). Furthermore, since there is a lack of CMV knowledge in our population, educating and effective counseling by obstetricians/ gynecologists to the pregnant women are recommended.

## INTRODUCTION

*Cytomegalovirus* (CMV) is a member of the *Herpesvirus* family, and constitutes the most common viral cause of congenital infections varying from asymptomatic state to fatal disease form in newborns (1). Approximately between 2 to 22 newborns per 1000 live births are congenitally infected with CMV, depending on variety of socioeconomic backgrounds and ethnic groups of different populations in the world (2).

Although most children with congenital CMV (cCMV) infection are normal or asymptomatic, however about 2% of asymptomatic infants and about half of the infants with CMV symptoms at birth (5–10% of total infected infants) will develop long term sequelae because of the infection (3). In other words, the lack of neonatal CMV screening program leads to identification of merely a small proportion of children with clinical symptoms suspicious to cCMV infection (4).

Based on recent investigations, diagnosis of infants with cCMV infection at early stages, when associated with useful interventions, could prevent from the late onset sequelae of infection such as speech disabilities in children with bilateral hearing loss (5). However, prenatal CMV infection in neonates is commonly occurred after birth and accurate diagnosis of cCMV is only possible within the first three weeks of infants’ life (6).

In the last decade, traditional time-consuming detection methods of cCMV infection like culture assay have been replaced with fast-sensitive molecular tests such as PCR assays. Additionally, a great deal of studies has addressed the diagnostic feasibility of dried blood spots (DBS) on Guthrie cards by testing for CMV DNA in the light of its reliable sensitivity and specificity (7, 8). Unfortunately, limited data exsists on cCMV infection rate among Iranian neonates in which the frequency of cCMV infections are mostly reported based on serological evaluation of neonates.

The aim of this multi-center study was to investigate the prevalence of cCMV infection in the six cities of Tehran province, Iran, by testing infants’ Guthrie cards.

## MATERIALS AND METHODS

### Population study.

This prospective study was carried out at the seven university affiliated healthcare centers in the western regions of Tehran province from July 1 to August 31, 2017. These centers serve as the main referral units for neonatal screening of metabolic disorders in province, which approximately cover more than 70,000 newborns per year and present a wide range of cares and facilities to about 5 million people living in six cities in these regions.

Ethical committee of Iran University of Medical Sciences approved this project (ethical code: IR.IUMS.REC.1394.25708) and written informed consent was signed by parents of all study participants. Moreover, educational brochures with data on CMV transmission, infection and prevention were distributed among all parents. The main characteristics details and clinical data of infants were collected by questionnaire or from medical records and their maternal data were obtained from “electronic health documents of Iranian families” (SIB System). Hospitalized infants in neonatal intensive care unit (NICU) were excluded from the study.

### Sampling.

After completion of metabolic tests in the central reference laboratory, 2 circles from each Guthrie card (Whatman 903, LOT 82509/112) were cut out by sterile scissors, stored in sealed plastic bags containing desiccant (9) and transported to the research laboratory at institute of immunology and infectious diseases (Hazrate-Rasool hospital), for CMV PCR test.

### DNA extraction.

Total DNAs were extracted from DBS samples in triplicate by using thermal shock (modified protocol) as described by de Vries et al. (10). In brief, a disk of each DBS (3 mm in diameter) was punched into a 1.5 ml tube containing 35 μl Minimal Essential Medium (MEM, Sigma Aldrich Co.) and incubated at 4°C overnight. Samples were heated in thermal cycler (Labcycler Basic, Goettingen, Germany), according to the following program: 55°C for 60 min, 100°C for 7 min, 4°C for 2 min, and further centrifuged at 3300 ×g for 15 min. The supernatant (20–25 μl) was transferred to another tube and frozen at −80°C for at least one night.

### CMV DNA amplification.

An in-house nested PCR assay was developed with specific primers for amplification of a conserved region in the *gB* gene (glycoprotein B) of CMV. Briefly, using 7 μl of DNA template in a final volume of 25 μl PCR mixture consisted of 12.5 μl PCR Master Mix, 2 μl external primers, 3.5 μl sterile water and over conditions as follow: 94°C 2 min; [94°C 30 s, 58°C 45 s, 72°C 60 s] × 35 cycles; 72°C 5 min, a 450 bp fragment was amplified in the primary PCR. Subsequently, using 1 μl of first amplification product as template for the secondary PCR and under the same conditions as mentioned above, with except for primers (internal) and annealing temperature (55°C), a 220 bp fragment was amplified in the second round. Finally, PCR products were separated through electrophoresis on 2% agarose gels (Kowsar molecular Co. Iran) and visualized under the UV light. The sequences of oligonucleotides illustrated in Table 1.

**Table 1.** The Sequence of Primers used for nested PCR assay

**PCR**	**Primers**	**Sequence (5′-3′)**	**Product Size**
Conventional n-PCR (*gB* Gene)	Outer-1	ACAGACACAAACAGCACCCA	450 bp
	Outer-2	TAAGGTGACGACAGGTTGGC	
	Inner-1	ACACGCATACCTCAACACC	220 bp
	Inner-2	GGCCCATGGTTCCGAAGCG	
Internal Control (β-globin Gene)	PC03	ACACAACTGTGTTCACTAGC	110 bp
	PC04	CAACTTCATCCACGTTTCACC	

### Quality Control.

A blank Whatman card was punched before testing each sample to prevent cross contamination between DBSs. CMV PCR assay was set up using triplicate testing of DBS spotted with different dilutions of CMV positive whole blood samples obtained from 7 infants with congenital CMV infection. It was also set up for important parameters (e.g. annealing temperatures, cycling numbers, and primer concentrations) to avoid false negative and positive results. Additionally, a fragment of human β-globin gene was amplified as internal control with previously published primers by Saiki et al. (PC03/PC04), to validate amplification reactions (11). DBS samples with positive results in at least 2 of 3 DBS punches were counted as CMV positive (10) and cCMV infection in newborns with CMV positive cards was confirmed through positive urine PCR tests.

### Clinical evaluation of infants.

All parents were informed of CMV screening result on their infants. In addition, sufficient data about CMV infection and its consequences in newborns were given specifically to the parents of infected infants. Furthermore, a pediatrician examined their infants and necessary laboratory tests such as full blood count and liver function test were conducted for them at the hospital.

### Data Analysis.

Univariate analyses were performed using the Fisher’s exact test and chi-squared test, to compare background variables between two groups of CMV infected and non-infected infants. Statistical analyses were performed using MedCalc for Windows, version 18.11 (MedCalc Software, Ostend, Belgium). P-value <0.05 was considered as significant.

## RESULTS

In total, 1200 newborns were recruited for screening of cCMV infection (about 10% of eligible infants), of which 26 were excluded due to different problems such as inappropriate sampling and/or missing data. In highlights, 673 were males and 501 were females, the mean birth weight of infants at birth time was 3088 gr. (SD= ± 352), preterm delivery was documented in 145 cases, and symptoms associated with cCMV infection (petechiae, jaundice and hearing loss) were observed in 168 newborns.

According to results of CMV PCR test, CMV DNA was found in DBS and urine samples of 4 infants (0.34%, Confidence Interval 95% (CI): 0.09–0.87), which approximately is equal to 3 cases per 1000 live births (1 per 294). In details, all four infants with cCMV infection (1 male and 3 female) were asymptomatic at birth and no symptoms or signs was observed in general examination. Their results of clinical and laboratory assessment revealed a normal condition similar to other children. Anti-CMV IgG antibody was found in the serum samples of all cases while anti-CMV IgM antibody was detected in only one infected infant.

In statistical analysis of data, no factor was found in association with neonatal CMV infection. The main characteristics data of infants by cCMV infection status demonstrated in Table 2.

**Table 2.** Analysis of demographic and clinical characteristics of infants by cCMV infection status

**Infants**	**CMV-Positive (%)**	**CMV-Negative (%)**	**P-Value**
Sex			NS^**^
Male	1 (0.2)	672 (99.8)	
Female	3 (0.6)	498 (99.4)	
Preterm birth (<37 weeks)			NS
Yes	2 (1.3)	143 (98.7)	
No	2 (0.2)	1027 (99.8)	
Jaundice			NS
Yes	1 (0.8)	113 (99.2)	
No	3 (0.2)	1057 (99.8)	
Petechiae			NS
Yes	0 (0.0)	43 (100.0)	
No	4 (0.3)	1127 (99.7)	
Hearing loss^*^			NS
Yes	0 (0.0)	11 (100.0)	
No	4 (0.3)	1159 (99.7)	
Birth weight (mean ± SD)	2975.0 (±206)	3201.7 (±499)	NS
Body length (mean ± SD)	47.0 (±0.8)	49.0 (±3.0)	NS
Head circumference (mean ± SD)	34.0 (±0.8)	35 (±2.0)	NS

* Results of initial hearing screening test by OAE test

** Not Significant

### Maternal factors.

The mean age of infants’ mothers at delivery time was 27.8 years (range: 16–45 years). Results of CMV serological test (on first trimester) were available for 973 women, of which, more than 95% had anti-CMV IgG antibody (previous immunity) in their serums. CMV awareness level was very low in our population and Thirty-two women have heard CMV before this project (2.7%). No maternal factors were found statistically associated with congenital CMV infection in newborns.

## DISCUSSION

While congenital CMV infection is known as a public health priority in developed countries, context is not clear in developing countries, like Iran (12). The current study provides documented data for the first time in Iran on congenital CMV infection rate by testing infants’ DBS cards. According to our results, cCMV infection was observed in 4 of 1176 infants who born in 6 cities of Tehran province, Iran (0.34%).

Our finding in this study is consistent with previous report from Tehran in which 0.32% of neonates (2 of 620) in a hospital were infected with CMV at birth (13). Additionally, similar findings were reported by two other studies with large sample sizes from Iran in which 0.49% and 0.65% of newborns in Isfahan and Gorgan cities (central and northern regions of Iran) were infected with CMV, respectively (14, 15).

In comparison with developing countries with high maternal CMV seroprevalence, the frequency of cCMV infection that we found in the current study is in agreement with recently reported rates of infection from Brazil (0.6%) and China (0.7%), but in contrast to one report from Turkey (1.9%) (16–18). Differences in socioeconomic status and public health levels as well as ethnicity variations within populations, probably justify some of these reported differences between studies (19).

All infected neonates with CMV were born asymptomatic in our study (A mild jaundice in one case was observed). Consistently, it has been reported by many studies from populations of countries with high maternal CMV seroprevalence (20). One reason may be due to existing of maternal antibodies in neonates (anti-CMV IgG) which pass during fetal periods from mother-to-child. Approximately 95% of neonates in our study were born from mothers with anti-CMV IgG antibody in their serum. In addition, many researchers previously shown that anti-CMV IgG antibodies were found positive in the serum samples of 85% to 100% of Iranian pregnant women (21–26).

According to previous studies, more than 85% of CMV infected infants will never develop the late onset sequelae of infection. Additionally, it is indicated by some reports that positive DBS cards may potentially discriminate infants at higher risk for developing sequelae (27). In line with these reports, a chorioretinitis was detected in one of four infected infants in current study after 9 months of birth (data not shown).

Currently, while an efficient vaccine for cCMV infection is not available, risk of infection among populations can be reduced by developing prevention-based strategies like attention to simple hygiene practices such as routine hand washing (28). In this study, few parents have heard CMV or CMV associated sequelae before and awareness level about CMV precautionary actions was very low in our population.

At last, there were some limitations in this study. Firstly, number of infected infants was not sufficient for the accurately analysis of results and secondly, the study site was located in one province and results may not be generalizable to other regions of our country.

## CONCLUSION

This survey revealed that congenital CMV infection in neonates is at least twice more prevalent than metabolic and endocrine disorders (1 in 950) which are currently included in the screening panel of neonatal diseases in Iran (29). Therefore, at least screening of symptomatic infants at birth seems to be necessary. In addition, educating the females at childbearing ages by healthcare-providers and effective counseling of pregnant women by obstetricians/ gynecologists are needed. Moreover, further studies with larger sample sizes in different regions of our country are recommended.
